# ﻿Crab spiders (Araneae, Thomisidae) of Jinggang Mountain National Nature Reserve, Jiangxi Province, China

**DOI:** 10.3897/zookeys.1095.72829

**Published:** 2022-04-13

**Authors:** Ke-Ke Liu, Yuan-hao Ying, Alexander A. Fomichev, Dan-chen Zhao, Wen-hui Li, Yong-hong Xiao, Xiang Xu

**Affiliations:** 1 College of Life Science, Jinggangshan University, Ji’an 343009, Jiangxi, China; 2 Altai State University, Lenina Pr., 61, Barnaul, RF-656049, Russia; 3 Tomsk State University, Lenina Pr., 36, Tomsk, RF-634050, Russia; 4 Key Laboratory of Agricultural Environmental Pollution Prevention and Control in Red Soil Hilly Region of Jiangxi Province, Jinggangshan University, Ji’an, 343009, Jiangxi, China; 5 College of Life Science, Hunan Normal University, Changsha 410081, Hunan, China

**Keywords:** Aranei, biodiversity, distribution, East Asia, new record, new species, taxonomy

## Abstract

A list of 34 thomisid species belonging to 21 genera collected in Jangxi Province of China is provided. Five new species are described: *Angaeusxieluae* Liu, **sp. nov.** (♂♀), *Lysitelessubspirellus* Liu, **sp. nov.** (♀), *Oxytatemucunica* Liu, **sp. nov.** (♀), *Phartalingxiufengica* Liu, **sp. nov.** (♀), *Stephanopisxiangzhouica* Liu, **sp. nov.** (♀). A new combination is proposed: *Ebelingiaforcipata* (Song & Zhu, 1993) **comb. nov.** (ex. *Ebrechtella* Dahl, 1907). Previously unknown females of *E.forcipata* (Song & Zhu, 1993), *Oxytatebicornis* Liu, Liu & Xu, 2017, and *Xysticuslesserti* Schenkel, 1963 are described for the first time. *Stephanopis* O Pickard-Cambridge, 1869, a genus previously known from Australasia and South America, is recorded from the Asian mainland for the first time.

## ﻿Introduction

Thomisidae Sundevall, 1833, commonly known as crab spiders, is the seventh largest spider family with a global distribution, comprising 2154 extant species belonging to 171 genera (WSC 2022). More than half of the Thomisidae species are known from a single sex: 752 of these were described from females and 337 from males (WSC 2022). More than fifty species were described from juveniles (WSC 2022).

The family has never been globally revised, but regional revisions have been made in, e.g., Canada (Dondale and Redner 1978), Japan (Ono 2009), and China (Song et al. 1999), etc. In the past ten years, many efforts were made to review or re-assign species described or recorded from China. Although there are several recent publications dealing with revisions, re-assignments of species, and descriptions of unknown sexes of Chinese crab spiders, there are still many species requiring study.

Thomisids in China are relatively well studied due to the revisions by Song et al. (1999) and Tang and Li (2010a, b). Currently, 306 species belonging to 51 genera are known from this country (Li 2020; WSC 2022). Level of knowledge is uneven in different provinces. Numbers of species known per province varies from north (Heilongjiang, *n* = 10) to south (Hainan, *n* = 47). One of the poorly studied provinces is Jiangxi, with only eight known species (Li and Lin 2016). To fill this gap, we studied material collected from the Jinggang Mountain National Nature Reserve in Jiangxi Province of China.

The aims of the present paper are (1) to report findings of 34 species belonging to 21 genera, (2) to provide detailed descriptions of five new species, (3) to provide descriptions of previously unknown females of three species, (4) to propose a new combination, and (5) to provide the first record of the genus *Stephanopis* from Asian mainland.

## ﻿Materials and methods

More than 300 adult specimens belonging to 34 species from 21 genera were collected from Jinggang Reserve. Specimens were examined using a Zeiss Stereo Discovery V12 stereomicroscope with a Zoom Microscope System. Both male palps and female copulatory organs were detached and examined in 75% ethanol, using a Zeiss Axio Scope A1 compound microscope with a KUY NICE CCD. For SEM photographs, specimens were dried under natural conditions and photographed with a ZEISS EVO LS15 scanning electron microscope. The epigynes were cleared in pancreatin. Specimens including detached male palps and epigynes were stored in 80% ethanol after examination. All the specimens treated in this work are deposited in the Animal Specimen Museum, Life Science of College, Jinggangshan University (**ASM-JGSU**).

Measurements were taken with the AxioVision software (SE64 Rel. 4.8.3) and are given in millimetres. Terminology of the male and female copulatory organs follows Benjamin (2011), Ramírez (2014), and Machado et al. (2019). Promarginal and retromarginal teeth on the chelicerae are given as the first, second, third, etc., and measured from the base of the fang to the distal groove.

Leg measurements are given as total length (femur, patella, tibia, metatarsus, tarsus). The abbreviations used in the text are:

**Eyes**

**ALE** anterior lateral eye;

**AME** anterior median eye;

**MOA** median ocular area;

**PLE** posterior lateral eye;

**PME** posterior median eye.

**Leg segments**

**Fe** femur;

**Mt** metatarsus;

**Pt** Patella;

**Ta** tarsus;

**Ti** tibia.

**Spination**

**d** dorsal;

**p** prolateral;

**r** retrolateral;

**v** ventral.

**Male palp**

**Mac** macroseta;

**BTA** basal tegular apophysis;

**C** conductor;

**E** embolus;

**Eb** base of the embolus;

**Gr** embolic groove;

**MA** median apophysis;

**RTA** retrolateral tibial apophysis;

**RTP** ridge-shaped tegular process;

**SD** sperm duct;

**T** tegulum;

**TR** tegular ridge;

**Tt** tutaculum;

**VTA** ventral tibial apophysis.

**Epigyne**

**AH** anterior hood;

**At** atrium;

**CD** copulatory duct;

**CO** copulatory openings;

**ET** epigynal teeth;

**FD** fertilisation duct;

**GA** glandular appendage;

**MS** membranous sac;

**P** lateral pocket;

**Se** septum;

**SP** spermatheca;

**SS** septal stem;

**TrR** transverse ridge of copulatory opening.

### ﻿Taxonomic survey

#### Family Thomisidae Sundevall, 1833

The known crab spider fauna of Jiangxi Province is complemented by 31 additional species belonging to 15 genera and now numbers 38 species in 21 genera. The full list of thomisid spiders recorded in this province is presented in Table 1, which follows the taxonomic accounts.

**Table 1.** List of Thomisidae species recorded in Jinggang Mountain National Nature Reserve. Genera recorded for the first time are marked with an asterisk (*).

Genus	Species	No. of ♂♂	No. of ♀♀	Total
*Alcimochthes* Simon, 1885 *	*A.limbatus* Simon, 1885	2	7	9
*Angaeus* Thorell, 1881 *	*A.liangweii* (Tang & Li, 2010)	0	4	4
	*A.xieluae* sp. nov.	2	1	3
*Borboropactus* Simon, 1884 *	*B.jiangyong* Yin et al., 2004	1	3	4
*Diaea* Thorell, 1869 *	*D.subdola* O. P.-Cambridge, 1885	8	2	10
*Ebelingia* Lehtinen, 2004*	*E.forcipata* (Song & Zhu, 1993) comb. nov.	3	5	8
*Ebrechtella* Dahl, 1907 *	*E.pseudovatia* (Schenkel, 1936)	1	2	3
	*E.tricuspidata* (Fabricius, 1775)	4	1	5
*Epidius* Thorell, 1877 *	*E.gongi* (Song & Kim, 1992)	2	5	7
*Indoxysticus* Benjamin & Jaleel, 2010 *	*I.tangi* Jin & Zhang, 2012	2	0	2
*Lysiteles* Simon, 1895 *	*L.minusculus* Song & Chai, 1990	1	0	1
	*L.silvanus* Ono, 1980	10	55	65
	*L.subspirellus* sp. nov.	0	2	2
*Misumenops* F. O. P.-Cambridge, 1900 *	*M.hunanensis* Yin, Peng & Kim, 2000	1	2	3
*Monaeses* Thorell, 1869	*M.aciculus* (Simon, 1903)	2	1	3
*Oxytate* L. Koch, 1878	*O.bicornis* Liu, Liu & Xu, 2017	1	2	3
	*O.forcipatus* Zhang & Yin, 1998	1	0	1
	*O.mucunica* sp. nov.	0	1	1
	*O.striatipes* L. Koch, 1878	4	0	4
*Ozyptila* Simon, 1864 *	*O.nipponica* Ono, 1985	1	0	1
*Pharta* Thorell, 1891 *	*P.brevipalpus* (Simon, 1903)	23	19	42
	*P.lingxiufengica* sp. nov.	0	1	1
*Smodicinodes* Ono, 1993 *	*S.hupingensis* Tang, Yin & Peng, 2004	0	1	1
*Stephanopis* O. Pickard-Cambridge, 1869 *	*S.xiangzhouica* sp. nov.	0	1	1
*Strigoplus* Simon, 1885 *	*S.guizhouensis* Song, 1990	1	2	3
*Synema* Simon, 1864	*S.zonatum* Tang & Song, 1988	0	1	1
*Thomisus* Walckenaer, 1805 *	*T.labefactus* Karsch, 1881	36	8	44
*Tmarus* Simon, 1875 *	*T.circinalis* Song & Chai, 1990	0	1	1
	*T.longqicus* Song & Zhu, 1993	3	4	7
*Xysticus* C. L. Koch, 1835	*X.croceus* Fox, 1937	26	8	34
	*X.hedini* Schenkel, 1936	2	0	2
	*X.kansuensis* (Tang, Song & Zhu, 1995)	0	2	2
	*X.kurilensis* Strand, 1907	5	5	10
	*X.lesserti* Schenkel, 1963	3	9	12

##### ﻿Genus Angaeus

Taxon classificationAnimaliaAraneaeThomisidae

Thorell, 1881

4CC8AFE9-C3CE-576F-8F6F-FAD96C74AA91

###### Comments.

This genus includes 11 species, mainly distributed in tropical Asia (India, Malaysia (Borneo), Myanmar, Vietnam, Singapore, and Indonesia) (WSC 2022). More than half of these species are recorded from China and have been revised by Benjamin (2013). It is worth mentioning that the female of the type species, *Angaeuspudicus* Thorell, 1881, remains unknown.

##### ﻿ Angaeus xieluae

Taxon classificationAnimaliaAraneaeThomisidae

Liu sp. nov.

E9FBDCC7-1167-52FB-9B12-1B2170F14A3A

http://zoobank.org/EADDA1BA-DD6D-4A83-9945-50116A0A90BD

Figs 1 , 2 , 3

###### Material examined.

***Holotype***: ♂, China: Jiangxi Province, Ji’an City, Jinggangshan County Level City, Jinggang Mountain National Nature Reserve, Eling Town, Tangnan Village, 26°43'8.4"N, 114°7'51.6"E, 289 m, 3.X.2015, K. Liu et al. leg. ***Paratypes***: 1 ♀, Longshi Town, Huishi Park, 26°42'32.4"N, 113°56'49.2"E, 242 m, 2.V.2015, K. Liu et al. leg.; 1 ♂, Eling Town, Shenyuan Village, 26°43'26.4"N, 114°7'44.4"E, 277 m, 28.V.2015, K. Liu et al. leg.; 1 ♂, Huangao Town, Zhongqiuba, 4.IV.2015, K. Liu et al. leg.

###### Etymology.

The specific name is a matronym in honour of Miss Xie Lu from the College of Life Science, Jinggangshan University, who helped us in Longshi Town, where the paratype of the new species was collected.

###### Diagnosis.

**Figure 1.**
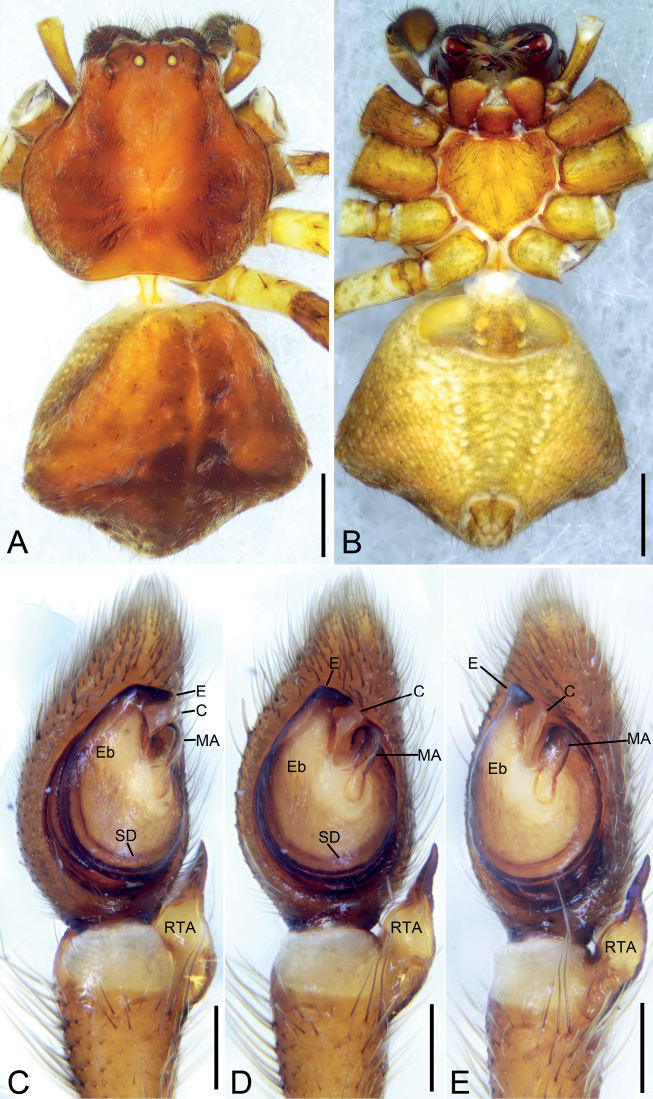
*Angaeusxieluae* sp. nov., male holotype. **A** habitus, dorsal view **B** same, ventral view **C** palp, prolateral view **D** same, ventral view **E** same, retrolateral view. Abbreviations: C – conductor, E – embolus, Eb – base of the embolus, MA – median apophysis, RTA – retrolateral tibial apophysis, SD – sperm duct. Scale bars: 0.5 mm (**A, B**), 0.1 mm (**C–E**).

**Figure 2.**
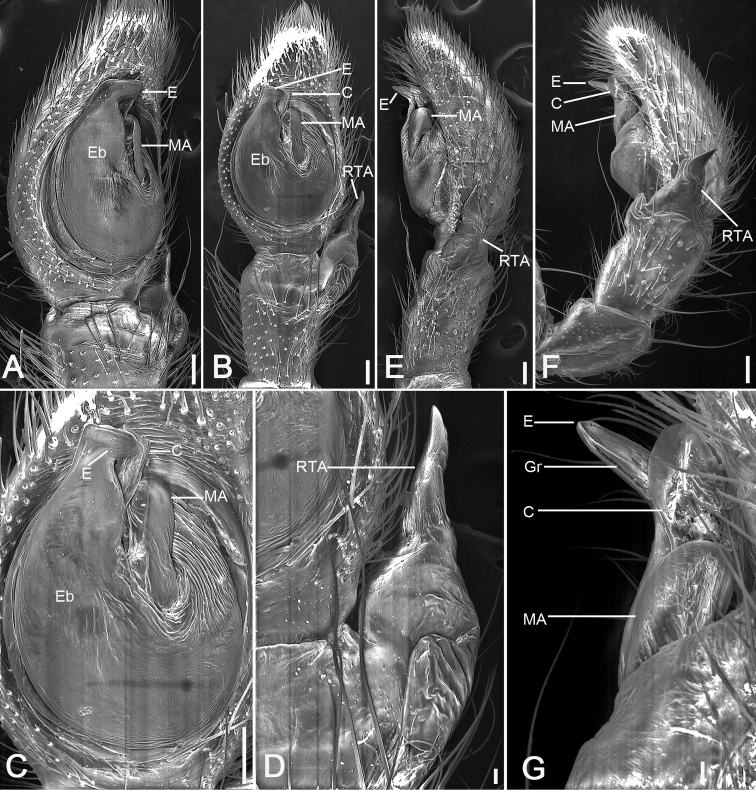
SEM micrographs of *Angaeusxieluae* sp. nov. male palp (holotype). **A** prolatero-ventral view **B** ventral view **C** same, details of bulb **D** same, details of retrolateral tibial apophysis **E** retrolateral view **F** retrolateral view, slightly dorsal **G** same, details of embolus. Abbreviations: C – conductor, E – embolus, Eb – base of the embolus, Gr – groove, MA – median apophysis, RTA – retrolateral tibial apophysis. Scale bars: 0.1 mm (**A–C, E, F**), 20 μm (**D, G**).

**Figure 3.**
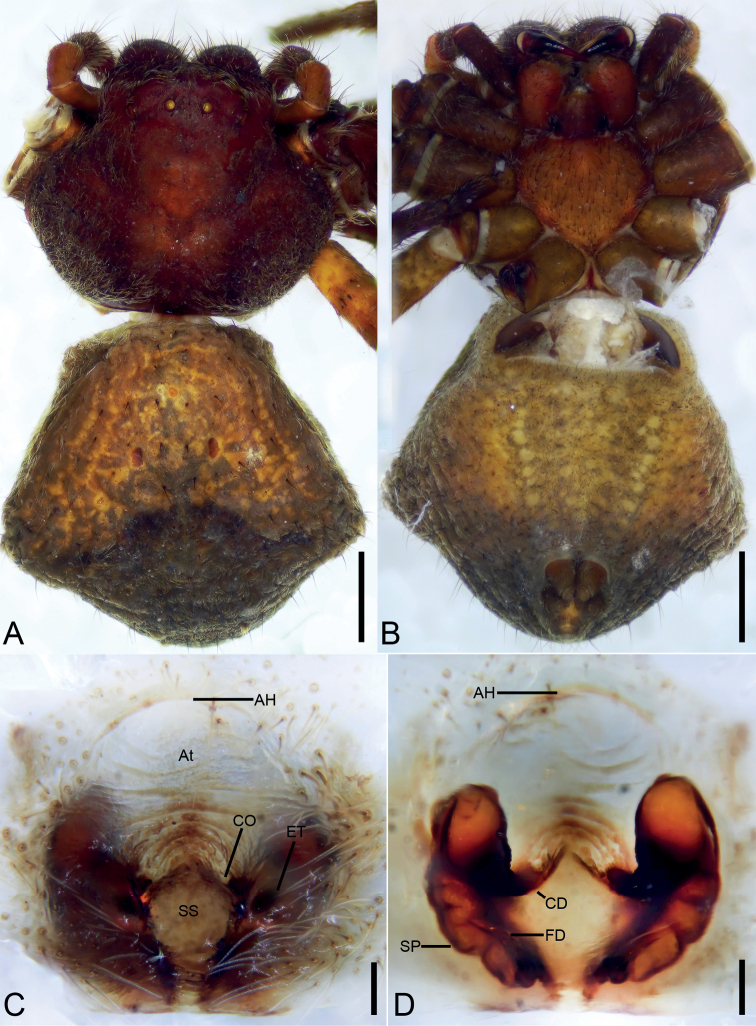
*Angaeusxieluae* sp. nov., female paratype. **A** habitus, dorsal view **B** same, ventral view **C** epigyne, ventral view **D** same, dorsal view. Abbreviations: AH – anterior hood, At – atrium, CD – copulatory duct, CO – copulatory opening, ET – epigynal teeth, FD – fertilisation duct, SP – spermatheca, SS – septal stem. Scale bars: 0.5 mm (**A, B**), 0.1 mm (**C, D**).

The male of the new species resembles those of *A.rhombifer* Thorell, 1890, widely distributed in South-East Asia, in having embolus (*E*) with widened tip and retrolateral tibial apophysis (*RTA*) as long as tibia but can be distinguished from it by having embolus with bill-shaped tip and widened base and the median apophysis (*MA*) shifted retrolaterally (vs. embolus with narrow base and the median apophysis starting from the centre of the tegulum) (cf. Figs 1D, 2B, C and Benjamin 2013: fig. 2A). The female of *A.xieluae* sp. nov. also resembles that of *A.rhombifer* in having elongated comma-shaped spermathecae (*SP*) but can be separated from the latter by reduced septal stem (*SS*) and arcuate anterior hood (*AH*) spaced from spermathecae (vs. well-developed septal stem and horizontal anterior hood adjoining to spermathecae) (cf. Fig. 3C, D and Benjamin 2013: fig. 2D, E).

###### Description.

**Male (*holotype*).** Habitus as in Fig. 1A, B. Total length 6.22. Carapace: 2.70 long, 3.11 wide, anteriorly narrowed to 1.6, with abundant fluffy setae. Eye sizes and interdistances: AME 0.09, ALE 0.22, PME 0.11, PLE 0.19, AME–AME 0.16, AME–ALE 0.14, PME–PME 0.28, PME–PLE 0.28, AME–PME 0.29, AME–PLE 0.54, ALE–ALE 0.64, PLE–PLE 1.0, ALE–PLE 0.26; MOA 0.45 long, front width 0.33, back width 0.51. Sternum (Fig. 1B) oval, with notch anteromedially. Abdomen (Fig. 1A, B): 3.58 long, 3.67 wide. Leg measurements: I 13.33 (4.22, 1.58, 3.88, 2.44, 1.21); II 13.56 (4.34, 1.59, 4.11, 2.38, 1.14); III 5.99 (1.86, 0.83, 1.48, 1.06, 0.76); IV 6.82 (2.15, 0.94, 1.57, 1.43, 0.73). Leg spination: I Fe: d4, p5, r4; Ti: p2, r2, v8; Mt: p2, r2, v6; II Fe: d10; Ti: p2, r2, v7; Mt: p3, r3, v4; III Fe: d2, p2, r2; Ti: p2, r1, v2; Mt: d2, v4; IV: Fe: d2; Ti: p2, r1, v2; Mt: d2, v2.

Colouration. Carapace reddish brown. Chelicerae dark brown. Endites and labium reddish brown. Sternum yellow. Legs yellow brown, with several dark spots near bases of setae; legs III and IV paler, with pale and dark colours. Abdomen reddish brown, medially with arch-shaped dark mark.

Palp (Figs 1C–E, 2). Femur 2 × longer than patella. Patella slightly shorter than tibia. Retrolateral tibial apophysis (*RTA*) large, as long as tibia, with thick and swollen base and ventral tibial apophysis, apex of RTA gradually pointed, directed dorsally. Cymbium drop-shaped, 2 × longer than wide. Tegulum oval, median apophysis (*MA*) spoon-shaped, extending from subcentre to 1 o’clock position, with a small hook-like apex. Sperm duct (*SD*) U-shaped, gradually tapering, arising at 1 o’clock position. Conductor (*C*) partly hidden by embolus, located close to the apex of median apophysis. Base of the embolus (*Eb*) ~ 3 × wider than the median apophysis. Embolus with clear sperm groove (*Gr*).

**Female.** Habitus as in Fig. 3A, B. Total length 8.07. Carapace: 3.56 long, 3.51 wide, anteriorly narrowed to 0.6 × of its maximum width. Eye sizes and interdistances: AME 0.08, ALE 0.19, PME 0.11, PLE 0.18, AME–AME 0.25, AME–ALE 0.15, PME–PME 0.29, PME–PLE 0.30, AME–PME 0.34, AME–PLE 0.52, ALE–ALE 0.71, PLE–PLE 1.00, ALE–PLE 0.23. MOA 0.56 long, front width 0.39, back width 0.52. Abdomen (Fig. 3A, B): 4.51 long, 4.16 wide, with abundant depressed patches. Leg measurements: I 11.99 (3.70, 1.42, 3.53, 2.20, 1.14); II 11.95 (3.78, 1.48, 3.44, 2.20, 1.05); III 5.81 (1.87, 0.78, 1.36, 1.10, 0.7); IV 6.74 (2.16, 0.86, 1.63, 1.34, 0.75). Leg spination: I Fe: d6, p1; Ti: p2, r2, v8; Mt: p3, r3, v4; II Fe: d6; Ti: p1, v2; Mt: p3, r3, v4; III Fe: d2; Ti: p1, v2; Mt: d2, p1, v4; IV: Fe: d2; Ti: p1, r1, v2; Mt: d2, p1, r1, v2.

Colouration as in Fig. 3A, B. Carapace reddish brown. Chelicerae dark brown. Endites and labium reddish brown. Sternum reddish brown. Legs yellow brown, with several dark spots near bases of setae; legs III and IV paler, with pale and dark colours. Abdomen yellow brown, subposteriorly with arch-shaped dark mark.

Epigyne (Fig. 3C, D). Anterior hood (*AH*) arcuate. Atrium (*At*) oval, 1.5 × wider than long, with several transverse wrinkles. Copulatory openings (*CO*) clearly visible, located within posteromedian part of the atrium. Epigynal teeth (*ET*) robust and blunt, inclined posteriorly. Septal stem (*SS*) round and convex. Spermathecae (*SP*) 2.5 × longer than wide, with several distinct constrictions, separated in the anterior part by 1.5 × their width and closely spaced in the posterior part. Copulatory ducts (*CD*) shorter than the spermathecae width. Fertilisation ducts (*FD*) nearly as long as 1/2 spermathecal length.

###### Distribution.

Known only from the type locality in Jiangxi Province of China (Fig. 17).

##### ﻿Genus Ebelingia

Taxon classificationAnimaliaAraneaeThomisidae

Lehtinen, 2004

C779A2A2-BDE3-5483-9D2A-E57F132ABE06

###### Comments.

This genus includes only two species, both from East Asia (WSC 2022).

##### ﻿ Ebelingia forcipata

Taxon classificationAnimaliaAraneaeThomisidae

(Song & Zhu, 1993) comb. nov.

86C3940C-4513-5183-A15E-6050529D8D4D

Figs 4 , 5 , 6

Misumenops forcipatus Song & Zhu, in Song, Zhu & Li, 1993: 879, fig. 50A–C (♂); Song and Zhu 1997: 139, fig. 99A–C (♂); Song et al. 1999: 482, fig. 279H (♂).
Ebrechtella forcipata : Lehtinen 2004: 165.

###### Material examined.

China: Jiangxi Province, Ji’an City, Jinggangshan County Level City, Jinggang Mountain National Nature Reserve: 1 ♂: Luofu Town, Changguling Forest Farm, 26°38'28"N, 114°14'6"E, 583 m, 5.X.2014, K. Liu et al. leg.; 1 ♀, Luofu Town, Pingtou Village, Changguling Forest Farm, road side, 26°39'18"N, 114°14'2.4"E, 438 m, 5.X.2014, K. Liu et al. leg.; 2 ♂, Ciping Town, Xingzhou Village, Baimukeng, 26°31'4.8"N, 114°11'9.6"E, 669 m, 3.X.2014, K. Liu et al. leg.; 1 ♀, Ciping Town, Xiazhuang Village, Zhushachong Forest Farm, 26°33'7.2"N, 114°11'27.6"E, 683 m, 4.X.2014, K. Liu et al. leg.; 2 ♀, Luofu Town, Pingtou Village, Tea forest, 26°38'14.4"N, 114°13'48"E, 419 m, 5.X.2014, K. Liu et al. leg.; 1 ♀, Huang’ao Town, Shantang Group, 26°28'22.8"N, 114°13'55.2"E, 315 m, 5.X.2015, K. Liu et al. leg.

###### Diagnosis.

**Figure 4.**
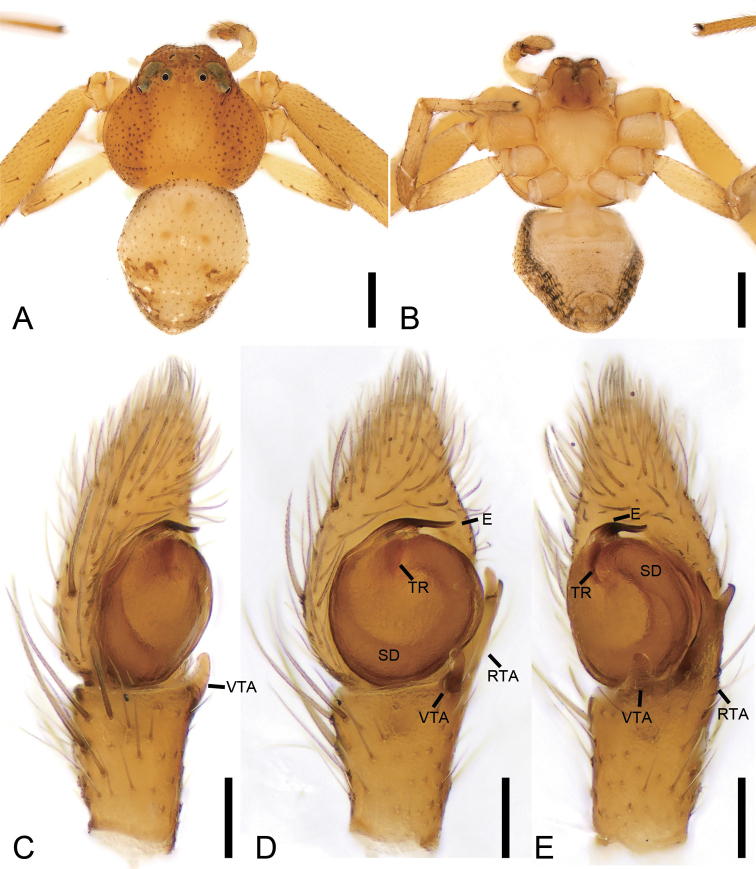
*Ebelingiaforcipata* (Song & Zhu, 1993), male. **A** habitus, dorsal view **B** same, ventral view **C** palp, prolateral view **D** same, ventral view **E** same, retrolateral view. Abbreviations: E – embolus, RTA – retrolateral tibial apophysis, SD – sperm duct, TR – tegular ridge, VTA – ventral tibial apophysis. Scale bars: 0.5 mm (**A, B**), 0.1 mm (**C–E**).

**Figure 5.**
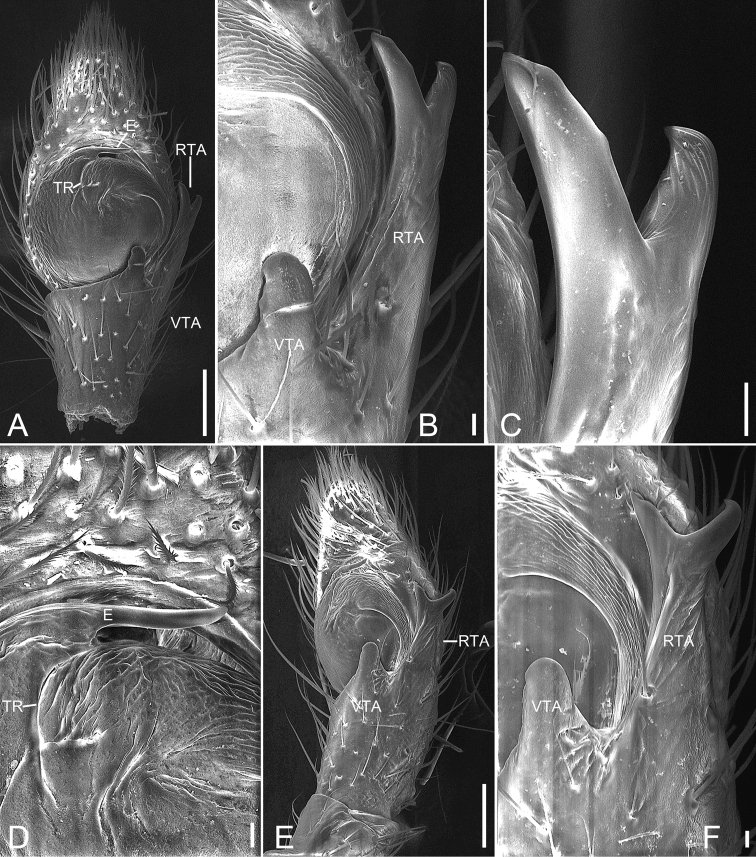
SEM micrographs of *Ebelingiaforcipata* (Song & Zhu, 1993) comb. nov., male palp. **A** ventral view **B** same, details of tibial apophysis **C** same, details of retrolateral tibial apophysis **D** same, details of embolus **E** retrolateral view **F** same, details of tibial apophyses. Abbreviations: E – embolus, RTA – retrolateral tibial apophysis, TR – tegular ridge, VTA – ventral tibial apophysis. Scale bars: 0.1 mm (**A, E**), 10 μm (**B, C, D, F**).

**Figure 6.**
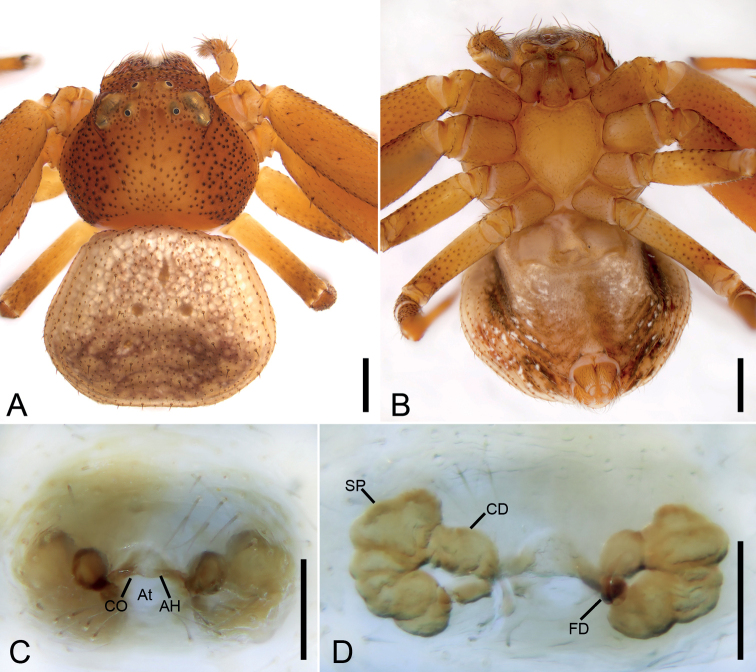
*Ebelingiaforcipata* (Song & Zhu, 1993), female. **A** habitus, dorsal view **B** same, ventral view **C** epigyne, ventral view **D** same, dorsal view. Abbreviations: AH – anterior hood, At – atrium, CD – copulatory duct, CO – copulatory opening, FD – fertilisation duct, SP – spermatheca. Scale bars: 0.5 mm (**A, B**), 0.1 mm (**C, D**).

The species differs from both congeners by the retrolateral tibial apophysis (*RTA*) with two equally long and thick branches (vs. dorsal branch much thinner and shorter than the ventral) (cf. Figs 4E, 5B, C, E, F; Song and Zhu 1997: fig. 100F; and Logunov 1992: fig. 9) in male and by thick and swollen copulatory ducts (*CD*) (vs. tube-shaped) (cf. Fig. 6D; Song and Zhu 1997: fig. 100D; and Kim and Gwon 2001: fig. 34) in female.

###### Description.

**Male.** Habitus as in Fig. 4A, B. Total length 2.60. Carapace: 1.35 long, 1.50 wide, with dense setae dorsally. Eye sizes and interdistances: AME 0.07, ALE 0.11, PME 0.06, PLE 0.08, AME–AME 0.16, AME–ALE 0.16, PME–PME 0.27, PME–PLE 0.21 AME–PME 0.13, AME–PLE 0.37, ALE–ALE 0.61, PLE–PLE 0.75, ALE–PLE 0.15. MOA 0.24 long, front width 0.29, back width 0.40. Sternum (Fig. 4B) slightly wider than long, anteromedial margin procurved, lateral margins serrulate, posterior end blunt. Abdomen (Fig. 4A, B): 3.58 long, 3.67 wide, with dense setae dorsally. Leg measurements: I 7.28 (2.25, 0.83, 1.82, 1.58, 0.80); II 6.63 (2.11, 0.80, 1.75, 1.18, 0.79); III 2.83 (0.87, 0.44, 0.69, 0.50, 0.33); IV 2.80 (0.87, 0.38, 0.71, 0.51, 0.33). Leg spination: I Fe: d3, p4, r2; Pa: p1, r1; Ti: d2, r3, v6; Mt: p4, r4, v4; II Fe: d4, p1; Pa: p1, r1; Ti: d2, p3, r3, v4; Mt: p4, r4, v4; III Fe: d3, p1; Pa: d1; Ti: d2, p1, r1, v2; Mt: p2, r2, v2; IV: Fe: d3, p1; Pa: d2, p1, r1; Ti: d2, p2, r1, v1; Mt: p2, r1.

Colouration (Fig. 4A, B). Carapace reddish brown, medially with yellowish band. Chelicerae, endites, and labium reddish yellow. Sternum and legs yellow. Abdomen yellow, posteriorly with two pairs of irregular yellow brown stripes, posterior one larger, with several white spots.

Palp (Figs 4C–E, 5). Femur 2 × longer than patella. Patella shorter than tibia. Retrolateral tibial apophysis (*RTA*) large, almost as long as tibia, with a broad base and apex split into two branches. Ventral tibial apophysis (*VTA*) short and blunt. Cymbium oval, length/width ratio 1.7. Tegulum almost round, with tegular ridge (*TR*) at the 12 o’clock position. Sperm duct (*SD*) wide, encircles almost the whole tegulum. Embolus (*E*) short, originating from ~ 11 o’clock position, free part at 12 o’clock, free part as long as ventral branch of RTA.

**Female.** Habitus as in Fig. 6A, B. As in male, except as noted. Total length 3.69. Carapace: 1.65 long, 1.77 wide. Eye sizes and interdistances: AME 0.07, ALE 0.1, PME 0.05, PLE 0.10, AME–AME 0.2, AME–ALE 0.2, PME–PME 0.36, PME–PLE 0.25, AME–PME 0.23, AME–PLE 0.44, ALE–ALE 0.72, PLE–PLE 0.92, ALE–PLE 0.19. MOA 0.34 long, front width 0.32, back width 0.48. Abdomen (Fig. 6A, B): 2.05 long, 2.19 wide, with abundant depressed patches. Leg measurements: I 6.17 (1.99, 0.89, 1.44, 1.15, 0.7); II 6.21 (2.08, 0.9, 1.37, 1.15, 0.71); III 2.95 (0.95, 0.57, 0.64, 0.46, 0.33); IV 3.07 (1.08, 0.41, 0.72, 0.46, 0.40). Leg spination: I missing; II Fe: d1; Ti: d2, r1, v4; Mt: p3, r3, v10; III Fe: d1; Pa: d2; Ti: d2; Mt: d1; IV: Fe: d3; Ti: d1; Mt: p2.

Colouration (Fig. 6A, B). Carapace reddish brown, medially with yellowish band. Chelicerae, endites and labium reddish yellow. Sternum and legs yellow. Abdomen, with numerous guanine spots, subposteriorly with two mottled stripes on the dorsal side, venter with few white spots.

Epigyne (Fig. 6C, D), width/length ratio ~ 2.6. Atrium (*At*) small, 3 × shorter than epigynal plate, located posteriorly from bell-shaped anterior hood (*AH*). Anterior hood located in centre. Copulatory openings (*CO*) located at posterolateral part of anterior hood. Spermathecae (*SP*) C-shaped, with several clearly visible constrictions, separated by more than width of anterior hood. Copulatory ducts (*CD*) broad, slightly longer than wide. Fertilisation ducts (*FD*) short, directed anteriorly.

###### Distribution.

China: Jiangxi and Fujian Provinces (Song et al. 1993; present data).

###### Note.

The left leg I was lost when we reviewed the holotype after photography.

###### Comments.

This species clearly belongs to *Ebelingia* due to the markedly bifurcated retrolateral tibial apophysis, short embolus, broad anterior hood, and kidney-shaped spermathecae.

##### ﻿Genus Lysiteles

Taxon classificationAnimaliaAraneaeThomisidae

Simon, 1895

CF499BA8-C42A-536A-ABDB-DF4FD0E939EF

###### Comments.

This genus includes 60 species, mainly distributed in eastern Asia. Half of them are recorded from China, but there are still 13 species known only from females in China, and three from males. Most of them (ten species) are recorded from Yunnan Province. No species were recorded from Jiangxi Province.

##### ﻿ Lysiteles subspirellus

Taxon classificationAnimaliaAraneaeThomisidae

Liu sp. nov.

CABFE381-4C1B-5D50-90C4-3D3F565C74E6

http://zoobank.org/F49B4189-C5CC-48D4-AB90-B141DAF7745D

Fig. 7

###### Material examined.

***Holotype***: ♀, China, Jiangxi Province, Ji’an City, Jinggangshan County Level City, Jinggang Mountain National Nature Reserve, Ciping Town, Dajing Village, Jingzhu Mountain, 26°30'10.8"N, 114°5'16.8"E, 1085 m, 20.XII.2015, K. Liu et al. leg. ***Paratype***: 1♀, same data as for holotype, 26°29'42"N, 114°4'44.4"E, 1158 m, 13.VIII.2016, K. Liu et al. leg.

###### Etymology.

The specific name is derived from that of a similar species, *L.spirellus* Tang, Yin, Peng, Ubick & Griswold, 2008; adjective.

###### Diagnosis.

**Figure 7.**
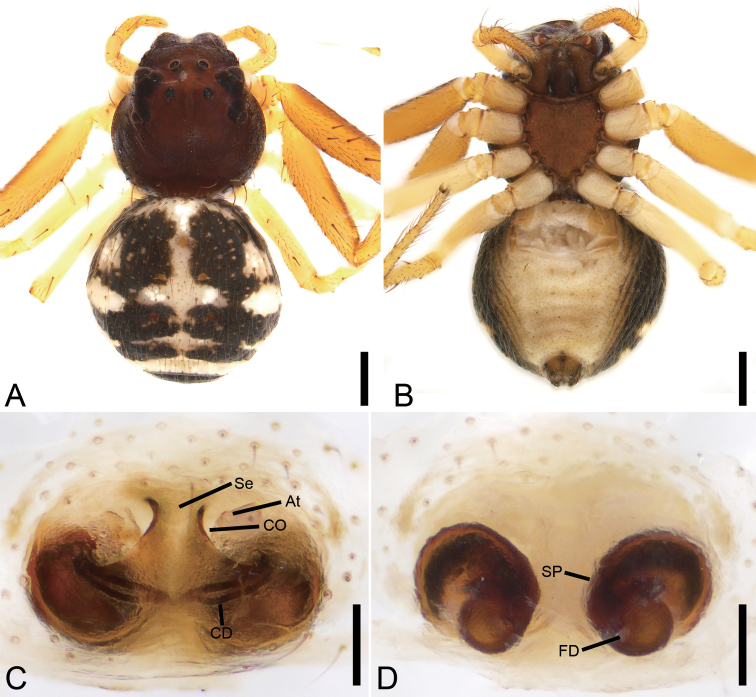
*Lysitelessubspirellus* sp. nov., female. **A** habitus, dorsal view **B** same, ventral view **C** epigyne, ventral view **D** same, dorsal view. Abbreviations: At – atrium, CD – copulatory duct, CO – copulatory opening, FD – fertilisation duct, Se –septum, SP – spermatheca. Scale bars: 0.5 mm (**A, B**), 0.1 mm (**C, D**).

The new species is similar to *L.auriculatus* Tang, Yin, Peng, Ubick & Griswold, 2008 and *L.spirellus* Tang, Yin, Peng, Ubick & Griswold, 2008 in having coiling spermathecae (*SP*), but differs from them by the carapace lacking pale median band (vs. present), abdomen with three pairs of large, touching, dark brown markings (Fig. 7A) (vs. relatively narrowed and widely separated markings). The new species can be distinguished from *L.auriculatus* by copulatory ducts (*CD*) located at posteromedian part of epigyne (vs. located anteriorly) (cf. Fig. 7C and Tang et al. 2008: fig. 2b, d). Finally, *L.subspirellus* sp. nov. differs from *L.spirellus* by spermathecae forming a tight coil (vs. loose coil) (cf. Fig. 7D and Tang et al. 2008: fig. 16d, f).

###### Description.

Habitus as in Fig. 7A, B. Total length 3.64. Carapace: 1.60 long, 1.49 wide, with several long setae around eye area and sublateral part of carapace. Eye sizes and interdistances: AME 0.12, ALE 0.19, PME 0.08, PLE 0.15, AME–AME 0.17, AME–ALE 0.17, PME–PME 0.30, PME–PLE 0.3 AME–PME 0.22, AME–PLE 0.17, ALE–ALE 0.71, PLE–PLE 0.87, ALE–PLE 0.26. MOA 0.37 long, front width 0.37, back width 0.47. Chelicerae with two promarginal (proximal larger, distal very small, nearly 1/3 × size of proximal one) and one (very small) retromarginal tooth. Sternum (Fig. 7B) longer than wide, anteromedial margin procurved, lateral margins serrulate, intercoxal triangles long, almost joining carapace, posterior end arch-shaped. Abdomen (Fig. 7A, B): 2.10 long, 1.81 wide, with abundant slender setae dorsally. Leg measurements: I 5.34 (1.62, 0.60, 1.35, 1.14, 0.63); II 5.52 (1.67, 0.67, 1.52, 1.02, 0.64); III 3.71 (1.18, 0.44, 0.97, 0.64, 0.48); IV 3.63 (1.19, 0.38, 0.88, 0.76, 0.42). Leg spination: I Fe: d2, p4; Pa: d1; Ti: d2, p4, r3, v4; Mt: p3, r3, v6; II Fe: d3, p1; Pa: d2, p1, r1; Ti: d1, p3, r2, v3; Mt: p3, r3, v4; III Fe: d3; Pa: d1; Ti: d2, p2, r1, v1; Mt: p2, r2, v1; IV: Fe: d2; Pa: d2; Ti: d3, p2, r2, v1; Mt: d2, p1, r1, v1.

Colouration (Fig. 7A, B). Carapace reddish brown. Chelicerae, endites, and sternum reddish brown. Labium dark reddish brown. Abdomen pale white, with three pairs of large dark brown stripes, anterior one irregular, others transverse, medially with paired white guanine spots, posteriorly with a semi-oval dark brown stripe; venter with two rows of yellow spots medially.

Epigyne (Fig. 7C, D). Epigyne 1.8 × wider than long. Anteromedian part with septum (*Se*) dividing atrium (*At*) into two oval parts. Copulatory openings (*CO*) located at posterior part of the fovea. Copulatory ducts (*CD*) almost straight, same length as spermathecal width. Spermathecae (*SP*) anticlockwise coiled, forming one full turn. Fertilisation duct (*FD*) shorter than spermathecal wide, directed anteriorly.

**Male.** unknown.

###### Comments.

At present, *L.digitatus* Zhang, Zhu & Tso, 2006, *L.distortus* Tang, Yin, Peng, Ubick & Griswold, 2008, and *L.torsivus* Zhang, Zhu & Tso, 2006 are known only from the males in mainland China; therefore, the new species may be conspecific with one of these three species.

###### Distribution.

Known only from the type locality in Jiangxi Province of China (Fig. 17).

##### ﻿Genus Oxytate

Taxon classificationAnimaliaAraneaeThomisidae

L. Koch, 1878

A576170F-6698-5759-BC94-DBED35ED154B

###### Comments.

This genus includes 28 species distributed mainly in Asia and Africa. Half of them have been recorded from China, mainly in Yunnan and Guangxi provinces (Li and Lin 2016), but two species are known only from females and three from males.

##### ﻿ Oxytate bicornis

Taxon classificationAnimaliaAraneaeThomisidae

Liu, Liu & Xu, 2017

838F6C23-BF36-529F-A4BC-BAD5F10AA541

Figs 8 , 9

Oxytate bicornis Liu, Liu & Xu, 2017: 194, figs 1A–D, 2A–C (♂).

###### Material examined.

China: ***holotype*** ♂, Jiangxi Province, Ji’an City, Jinggangshan County Level City, Jinggang Mountain National Nature Reserve, Ciping Town, Dajing village, 26.566°N, 114.125°E, 922 m, 13.VII.2014, K. Liu et al. leg.; 2 ♀, other data as in holotype; 1 ♀, Longshi Town, Yuankou Village, 26°41'31.2"N, 113°57'10.8"E, 265 m, 1.V.2015, K. Liu et al. leg.; 1 ♀, Huang’ao Town, Shantang Group, 26°28'26.4"N, 114°13'58.8"E, 306 m, 5.IV.2015, K. Liu et al. leg.

###### Diagnosis.

**Figure 8.**
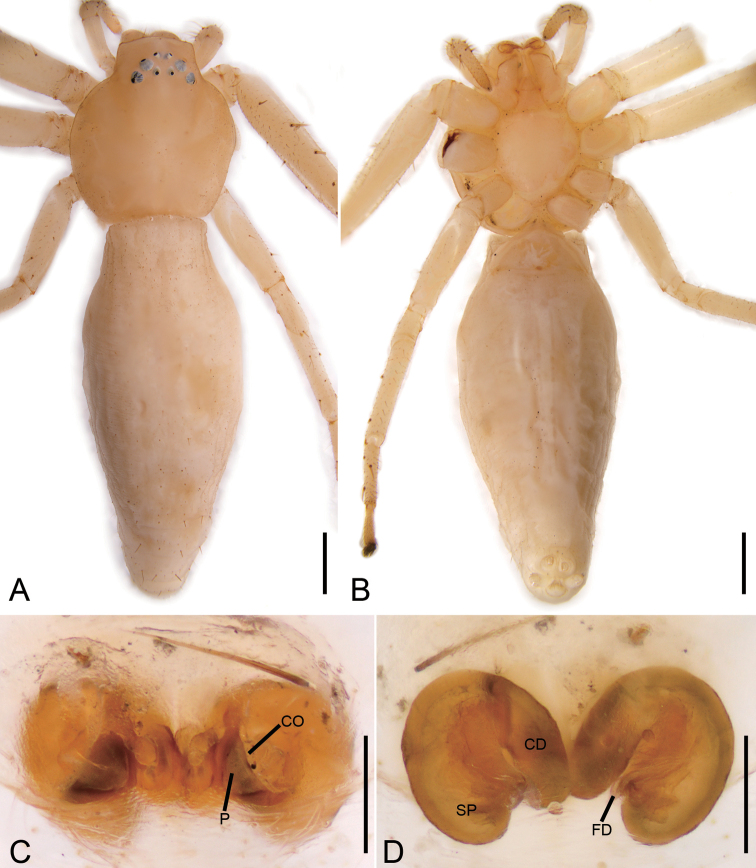
*Oxytatebicornis* Liu, Liu & Xu, 2017, female. **A** habitus, dorsal view **B** same, ventral view **C** epigyne, ventral view **D** same, dorsal view. Abbreviations: CD – copulatory duct, CO – copulatory opening, FD – fertilisation duct, P – pocket, SP – spermatheca. Scale bars: 0.5 mm (**A, B**), 0.1 mm (**C, D**).

**Figure 9.**
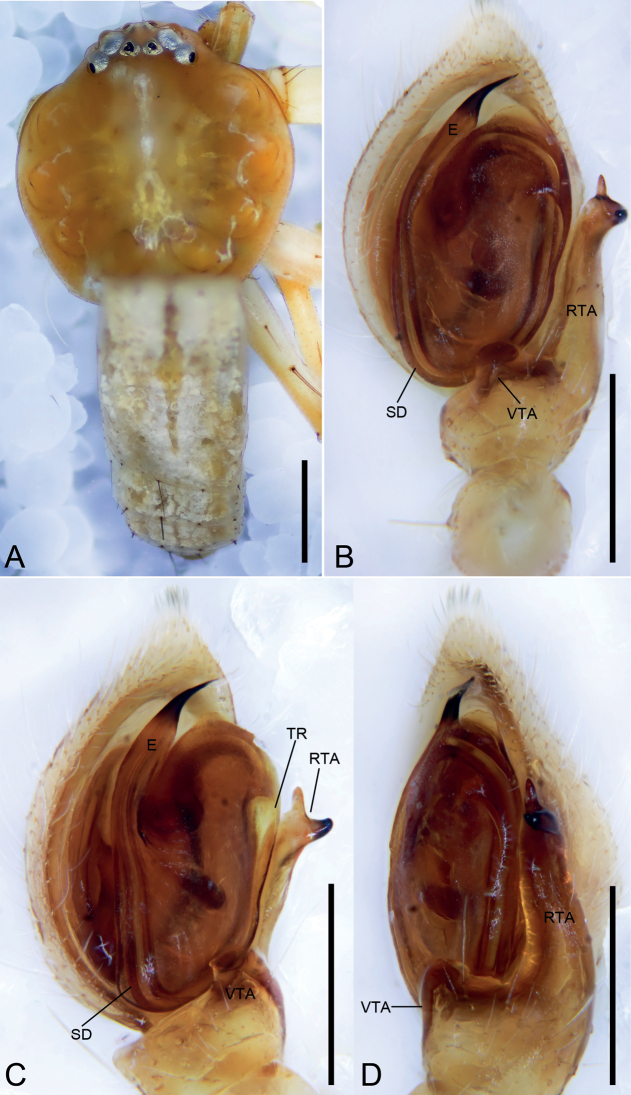
*Oxytatebicornis* Liu, Liu & Xu, 2017, male. **A** habitus, dorsal view **B** palp, ventral view **C** same, prolateral view **D** same, retrolateral view. Abbreviations: E – embolus, RTA – retrolateral tibial apophysis, SD – sperm duct, TR – tegular ridge, VTA – ventral tibial apophysis. Scale bars: 1 mm (**A**), 0.5 mm (**B–D**).

**Figure 10.**
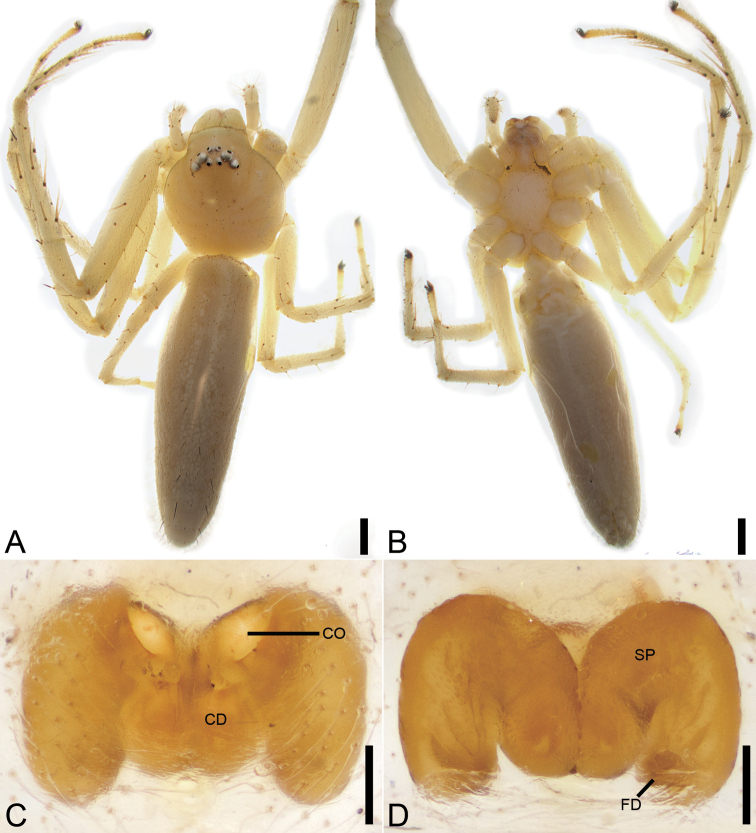
*Oxytatemucunica* sp. nov., female. **A** habitus, dorsal view **B** same, ventral view **C** epigyne, ventral view **D** same, dorsal view. Abbreviations: CD – copulatory duct, CO – copulatory opening, FD – fertilisation duct, SP – spermatheca. Scale bars: 0.5 mm (**A, B**), 0.1 mm (**C, D**).

Female of this species similar to that of *O.bhutanica* Ono, 2001 and *O.mucunica* sp. nov. in having vulva with M-shaped pattern formed by copulatory ducts (*CD*) and spermathecae (*SP*), but differs from both species by the copulatory openings (*CO*) oriented outwards (vs. inwards or anteriorly) and copulatory ducts (*CD*) 2 × thinner than spermathecae (*SP*) (vs. equal in size) (cf. Fig. 8C, D vs. Fig. 10C, D and Ono 2001: figs 4, 5). Male of *O.bicornis* resembles those of *O.bhutanica* in having spine-like embolus (*E*) and moderately long retrolateral tibial apophysis (*RTA*), reaching to the middle of the cymbium but can be distinguished by the bifurcated retrolateral tibial apophysis (vs. horn-like) (cf. Fig. 9B–D and Ono 2001: figs 2, 3).

###### Description.

**Female.** Habitus as in Fig. 8A, B. Total length 5.77. Carapace: 2.09 long, 2.02 wide. Eye sizes and interdistances: AME 0.08, ALE 0.11, PME 0.08, PLE 0.1, AME–AME 0.22, AME–ALE 0.16, PME–PME 0.22, PME–PLE 0.38, AME–PME 0.29, AME–PLE 0.58, ALE–ALE 0.66, PLE–PLE 1.08, ALE–PLE 0.31. MOA 0.42 long, front width 0.36, back width 0.38. Endites more than 2 × longer than wide. Sternum as in Fig. 8B with arch-shaped posterior end. Abdomen (Fig. 8A, B): 3.92 long, 1.11 wide, with regular transverse rows of strong spines on posterior part. Leg measurements: I 7.98 (2.45, 1.15, 2.15, 1.58, 0.65); II 7.79 (2.41, 1.10, 2.06, 1.50, 0.72); III 4.65 (1.39, 0.68, 1.27, 0.84, 0.47); IV 4.95 (1.76, 0.50, 1.25, 0.97, 0.47). Leg spination: I Fe: d4, p4, r1; Pa: d2, p1, r1; Ti: d2, p3, r3, v8; Mt: p2, r2, v6; II Fe: d4, p1, r1; Pa: d2, p1, r1; Ti: d3, p3, r3, v6; Mt: p2, r2, v6; III Fe: d2, p1; Pa: d2, p1; Ti: d1, p2, r1, v2; Met: p2, r2, v2; IV: Fe: d2; Pa: d2, p1, r1; Ti: d3, p2; Mt: p2.

Colouration. Carapace, chelicerae, endites, sternum, legs, and abdomen yellowish.

Epigyne (Fig. 8C, D). Epigyne 2 × wider than long. Copulatory openings (*CO*) hidden by lateral pockets (*P*). Spermathecae (*SP*) kidney-shaped, 2 × longer than wide. Copulatory ducts (*CD*) shorter than spermathecae, slightly curved, aggregative form V-shaped figure. Fertilisation ducts (*FD*) small, poorly visible and directed laterally.

**Male** (Fig. 9): See Liu et al. (2017: 194).

###### Comments.

Some females were collected in the type locality and others raised from juveniles. Newly collected females have general appearance and leg spination similar to the holotype male. Based on this, we consider them conspecific. This judgment will be confirmed or rejected in future when both sexes are collected together, simultaneously.

###### Distribution.

Known only from Jiangxi Province, China (Fig. 17).

##### ﻿ Oxytate mucunica

Taxon classificationAnimaliaAraneaeThomisidae

Liu sp. nov.

82082349-8748-5D4B-922D-3156A5B6C28F

http://zoobank.org/55A995BC-01C4-4586-B645-67A517D3D9CE

Fig. 10

###### Material examined.

***Holotype***: ♀, China, Jiangxi Province, Ji’an City, Jinggangshan City, Jinggang Mountain National Nature Reserve, Mucun Town, Guibian Village, 26°38'32.11"N, 113°53'51.99"E, 322 m, 31.VII.2019, K. Liu et al. leg.

###### Etymology.

The specific name is derived from the type locality, Mucun Town.

###### Diagnosis.

Female of the new species is similar to those of *O.bhutanica* Ono, 2001 and *O.bicornis* in having vulva with M-shaped pattern formed by copulatory ducts (*CD*) and spermathecae (*SP*), but differ from both species by the copulatory openings oriented anteriorly (vs. outwards or inwards) (cf. Fig. 10C vs. Fig. 9C and Ono 2001: fig. 4). Additionally, *O.mucunica* sp. nov. differs from *O.bicornis* by the copulatory ducts being as wide as spermathecae (vs. copulatory ducts 2 × thinner) (cf. Fig. 10D and Fig. 8D).

###### Description.

Habitus as in Fig. 10A, B. Total length 11.74. Carapace: 3.67 long, 3.33 wide. Eye sizes and interdistances: AME 0.08, ALE 0.14, PME 0.09, PLE 0.11, AME–AME 0.14, AME–ALE 0.07, PME–PME 0.08, PME–PLE 0.20, AME–PME 0.14, AME–PLE 0.39, ALE–ALE 0.39 PLE–PLE 0.77, ALE–PLE 0.05. MOA 0.52 long, front width 0.38, back width 0.43. Endites more than 2 × longer than wide, sub-trapezoidal. Abdomen (Fig. 10A, B): 8.42 long, 2.68 wide, with abundant white guanine spots dorsally and regular transverse rows of strong spines on posterior part. Leg measurements: I 8.56 (2.69, 0.99, 2.36, 1.65, 0.87); II 8.06 (2.50, 0.97, 2.24, 1.62, 0.73); III 4.77 (1.41, 0.60, 1.34, 0.90, 0.52); IV 4.88 (1.75, 0.54, 1.20, 0.93, 0.46). Leg spination: I Fe: d4 p4, r1; Pa: d2, p1, r1; Ti: d2, p3, r3, v8; Mt: p2, r2, v6; II Fe: d4, p1, r1; Pa: d2, p1, r1; Ti: d3, p3, r3, v6; Mt: p2, r2, v6; III Fe: d2, p1; Pa: d2, p1; Ti: d3, r2, v2; Mt: p2, r2, v2; IV: Fe: d2; Pa: d2, p1, r1; Ti: d2, p2; Mt: p2.

Colouration (Fig. 10A, B). Carapace, chelicerae, endites, sternum, legs, and abdomen yellowish.

Epigyne (Fig. 10C, D). Epigyne 1.5 × wider than long. Pockets absent. Copulatory openings (*CO*) large, as long as wide, with weakly sclerotised margins. Spermathecae (*SP*) cylindrical, smoothly merging into copulatory ducts. Copulatory ducts (*CD*) very wide, touching each other almost the entire length. Fertilisation ducts (*FD*) short, directed laterally.

**Male.** Unknown.

###### Notes.

While many juveniles were collected and reared in the lab, only one female reached maturity.

###### Comments.

The new species could potentially be a synonym of one of the species known only from males and occurring in China: *O.clavulata* Tang, Yin & Peng, 2008 (Yunnan) or *O.placentiformis* Wang, Chen & Zhang, 2012 (Guangxi).

###### Distribution.

Known only from the type locality in Jiangxi Province of China (Fig. 17).

##### ﻿Genus Pharta

Taxon classificationAnimaliaAraneaeThomisidae

Thorell, 1891

57276AA3-EFAB-59D2-AAEA-2249F508A66E

###### Comments.

This genus includes nine species, the majority of which are distributed in Southeast Asia (WSC 2022). Four species are known to occur in China, all in Yunnan and Guizhou provinces (Li and Lin 2016). Among them, only *P.tengchong* (Tang, Griswold & Yin, 2009) is known from the female.

##### ﻿ Pharta lingxiufengica

Taxon classificationAnimaliaAraneaeThomisidae

Liu sp. nov.

553FAD37-449F-5FB9-91C7-EFA9532009A6

http://zoobank.org/E2EB3363-9EEC-4BD7-82C4-65340A5F9CA3

Fig. 11

###### Material examined.

***Holotype***: ♀, China, Jiangxi Province, Ji’an City, Jinggangshan County Level City, Jinggang Mountain National Nature Reserve, Ciping Town, Dajing Village, Lingxiufeng Scenic Spot, 26°34'16.72"N, 114°07'00.56"E, 971 m, 1.X.2018, K. Liu et al. leg.

###### Etymology.

The specific name is derived from the type locality, Lingxiufeng Scenic Spot; adjective.

###### Diagnosis.

**Figure 11.**
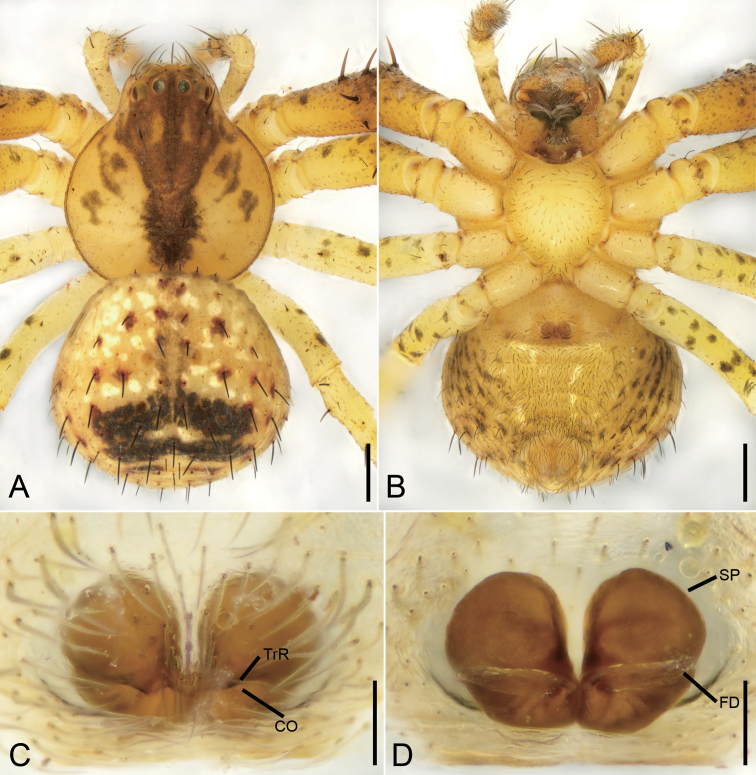
*Phartalingxiufengica* sp. nov., female holotype. **A** habitus, dorsal view **B** same, ventral view **C** epigyne, ventral view **D** same, dorsal view. Abbreviations: CO – copulatory opening, FD – fertilisation duct, SP – spermatheca, TrR – transverse ridge of copulatory openings. Scale bars: 0.5 mm (**A, B**), 0.1 mm (**C, D**).

The female of *P.lingxiufengica* is similar to that of *P.tangi* Wang, Mi & Peng, 2016 in having well-developed transverse ridge of copulatory openings (*TrR*) (= atrial intermediate margin of Wang et al. 2016) and invisible copulatory openings (*CO*). The new species can be easily differentiated from *P.tangi* by the convex transverse ridge of copulatory openings (vs. concave) (cf. Fig. 11C and Wang et al. 2016: fig. 2C).

###### Description.

Habitus as in Fig. 11A, B. Total length 3.08. Carapace: 3.67 long, 3.33 wide. Eye sizes and interdistances: AME 0.05, ALE 0.15, PME 0.10, PLE 0.14, AME–AME 0.07, AME–ALE 0.04, PME–PME 0.12, PME–PLE 0.13, AME–PME 0.22, AME–PLE 0.31, ALE–ALE 0.25, PLE–PLE 0.57, ALE–PLE 0.14. MOA 0.35 long, front width 0.18, back width 0.32. Chelicerae with three promarginal (proximal largest, distal smallest) and three retromarginal (middle and distal with a same base, distal largest, middle smallest) teeth. Endites slightly longer than wide, anterior part broad. Labium slightly wider than long. Sternum (Fig. 11B) with numerous setae, anteromedially procurved, with subtriangular posterior end. Abdomen (Fig. 11A, B): 1.69 long, 1.63 wide, with sparse, erected setae. Leg measurements: I 4.28 (1.35, 0.51, 1.22, 0.87, 0.33); II 4.09 (1.23, 0.48, 1.18, 0.80, 0.40); III 2.31 (0.73, 0.36, 0.59, 0.38, 0.25); IV 2.71 (0.93, 0.33, 0.64, 0.55, 0.26). Leg spination: I Fe: d4, p2; Pa: d2; Ti: d2, v10; Mt: v8; II Fe: d1; Pa: d2; Ti: d2, v10; Mt: v8; III Fe: d3; Pa: d1; Ti: d2, p2, v2; Mt: d2, v2; IV: Fe: d1; Pa: d1, r1; Ti: d3, p1, r1; Mt: d2.

Colouration (Fig. 11A, B). Carapace yellow-brown, medially with single broad, dark brown, mottled band. Legs yellow, basis of macrosetae on legs appearing as darkish brown dots. Chelicerae, endites, sternum, legs, and abdomen reddish yellow. Abdomen with sparse white guanine spots, medially with clear inverted T-shaped dark brown marking; macrosetal bases reddish brown; venter with pairs of longitudinal, short, guanine stripes.

Epigyne (Fig. 11C, D). Epigyne oval, 1.5 × wider than long, lacking hood. Copulatory openings (*CO*) invisible, hidden by transverse ridge of copulatory openings (*TrR*). Copulatory ducts (*CD*) not visible, possibly absent. Spermathecae (*SP*) oval, ~ 1.4 × longer than wide, anterior part of spermathecae slightly separated, posterior parts touching. Fertilisation ducts (*FD*) as long as width of spermatheca, directed laterally.

**Male.** unknown.

###### Comments.

There is only one species in the region known from the male only, *P.koponeni* Benjamin, 2014 (Thailand); however, it is unlikely to be conspecific with our female because of differences in colouration, and the long distance between type localities.

###### Distribution.

Known only from the type locality in Jiangxi Province of China (Fig. 17).

##### ﻿Genus Stephanopis

Taxon classificationAnimaliaAraneaeThomisidae

O. Pickard-Cambridge, 1869

4FEC636B-4AE2-594C-96B2-1B61D9F9043F

###### Comments.

This genus includes 23 species (WSC 2022). Most of them are distributed in South America, Oceania, Australia, and Papua New Guinea (WSC 2022). The recent phylogenetic analyses based on a matrix of 117 morphological characters scored for 77 terminal taxa have revealed a large variation in morphology among *Stephanopis* species (Machado and Teixeira 2021); these taxonomic revisions have greatly contributed to a better understanding of the group. Here we report the first species from the Asian continent as well as from the entire Oriental zoogeographical realm.

##### ﻿ Stephanopis xiangzhouica

Taxon classificationAnimaliaAraneaeThomisidae

Liu sp. nov.

EBFC9628-3C8F-5927-B124-69C424F328D2

http://zoobank.org/65E3AE25-DFA8-4629-9E0E-1EABD1AB4716

Figs 12 , 13

###### Material examined.

***Holotype***: ♀, China, Jiangxi Province, Ji’an City, Jinggangshan County Level City, Jinggang Mountain National Nature Reserve, Luofu Town, Xiangzhou Village, Fengshuping Group, 26°36'10.8"N, 114°15'28.8"E, 412 m, 5.VIII.2015, leg. K. Liu et al. leg.

###### Etymology.

The specific name refers to the type locality, Xiangzhou Village.

###### Diagnosis.

The new species is similar to *S.nigra* O. Pickard-Cambridge, 1869 by having slit-like copulatory openings (*CO*), but differs by lacking lateral sclerotised margins of copulatory openings (vs. lateral margins sclerotised), touching membranous sacs (vs. separated) and slightly separated spermathecae (vs. touching) (cf. Fig. 13 and Machado et al. 2019: fig. 37C, D).

###### Description.

**Figure 12.**
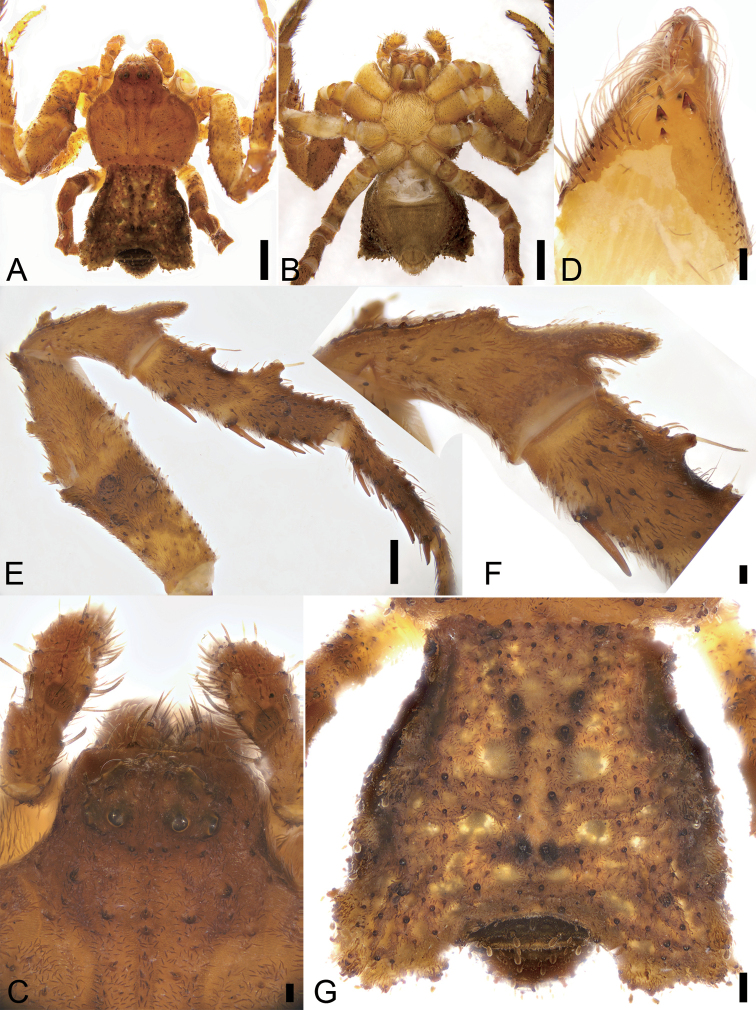
*Stephanopisxiangzhouica* sp. nov., female holotype. **A** habitus, dorsal view **B** same, ventral view **C** cephalic part, dorsal view **D** left chelicera, mesal view **E** left leg I, prolateral view **F** patella I, prolateral view **G** abdomen, dorsal view. Scale bars: 1 mm (**A, B**), 0.1 mm (**C, D, F**), 0.5 mm (**E, G**).

**Female.** Habitus as in Fig. 12A, B. Total length 5.48. Carapace: 2.78 long, 2.96 wide, covered with numerous strong, short, radially diverging setae and dense short plumose setae, with three rows of short strong setae along midline. Eye sizes and interdistances (Fig. 12C): AME 0.04, ALE 0.07, PME 0.05, PLE 0.06, AME–AME 0.07, AME–ALE 0.04, PME–PME 0.12, PME–PLE 0.13, AME–PME 0.22, AME–PLE 0.31, ALE–ALE 0.25, PLE–PLE 0.57, ALE–PLE 0.14. MOA 0.26 long, front width 0.13, back width 0.24. Chelicerae (Fig. D) with three promarginal (middle largest, distal and proximal smaller) and two retromarginal (proximal large, distal very small) teeth, and numerous macrosetae anteriorly. Endites 2 × longer than wide, ectal part without distinct constriction. Labium wider than long. Sternum (Fig. 12B) oval with short dense macrosetae. Abdomen (Fig. 12A, B, G): 2.79 long, 2.94 wide, pentagonal with pair of latero-posterior horns; dorsum covered with sparse brown clavate and small dense plumose setae. Leg measurements: I 5.52 (1.78, 0.82, 1.31, 1.14, 0.47); II 4.80 (1.74, 0.73, 1.08, 0.86, 0.39); III 3.18 (0.95, 0.50, 0.80, 0.51, 0.42); IV 3.30 (1.24, 0.46, 0.74, 0.50, 0.36). Femora, patellae, and tibiae of legs I and II with dorsal outgrowths, especially long in distal parts of patellae. Spination: I Fe: d1, p4, v4; Pa: d3; Ti: d2, v8; Mt: r1, v8; II Fe: d3, v4; Pa: d2; Ti: d2, v8; Mt: v6; IV: Ti: d1, r1.

Colouration as in Fig. 12. Carapace, chelicerae, endites, and labium reddish brown. Sternum yellow. Palpal tibia with one clear round dark brown patch. Legs yellow to dark brown, with numerous dark brown patches on femora and tibiae. Abdomen reddish brown, dorsally with numerous pale brown dots, without setae on those dots.

**Figure 13.**
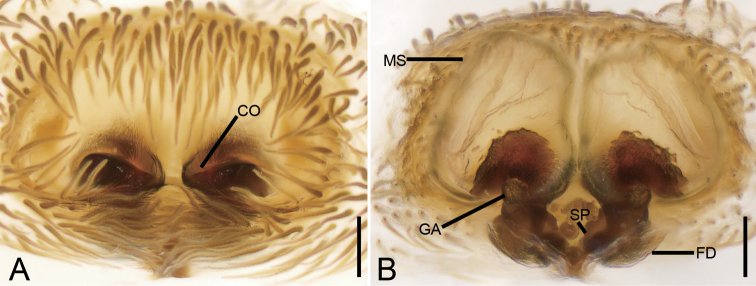
*Stephanopisxiangzhouica* sp. nov., female epigyne, holotype. **A** ventral view **B** dorsal view. Abbreviations: CO – copulatory opening, FD – fertilisation duct, GA – glandular appendage, MS – membranous sac, SP – spermatheca. Scale bars: 0.1 mm.

Epigyne (Fig. 13). Epigynal plate sub-trapezoidal, 1.3 × wider than long. Copulatory openings (*CO*) oriented horizontally separated by nearly 1/3 of their width. Membranous sacs (*MS*) transparent, located anteriorly, covering 2/3 of epigynal plate, touching each other. Glandular appendages of membranous sac (*GA*) spherical, short, as long as 1/3–1/2 width of spermatheca (*SP*). Spermathecae oval, slightly separated by 1/3 of its width. Fertilisation ducts (*FD*) gramineous leaf-shaped, as long as spermathecae, directed laterally.

**Male.** unknown.

###### Distribution.

Known only from the type locality in Jiangxi Province of China (Fig. 17).

##### ﻿Genus Xysticus

Taxon classificationAnimaliaAraneaeThomisidae

C. L. Koch, 1835

767686A9-604A-5FB2-A1C7-2E6B6A4F5416

###### Comments.

*Xysticus* is one of the most diverse genera in Thomisidae with 293 named species (WSC 2022). Fifty-nine species are known from China. Of these, 19 are known only from females, and five from males. Most species are distributed in northern China and only one species, *Xysticuslesserti* Schenkel, 1963, is known from Guizhou Province (Li and Lin 2016).

##### ﻿ Xysticus lesserti

Taxon classificationAnimaliaAraneaeThomisidae

Schenkel, 1963

FC3EB2E1-AD4E-5154-90EF-4BAF210FB38B

Figs 14 , 15 , 16

Xysticus lesserti Schenkel, 1963: 219, fig. 124a–c (♂); Marusik and Omelko 2014: 280, figs 15–17, 24 (♂).

###### Material examined.

China, Jiangxi Province: 1 ♀, Ji’an City, Jinggangshan County Level City, Jinggang Mountain National Nature Reserve, Ciping Town, Xiazhuang Village, Zhushachong Forest Farm, 26°33'3.6"N, 114°11'20.4"E, 630 m, 4.X.2014, K. Liu et al. leg.; 1 ♀, Ciping Town, Tongmuling, 26°37'12"N, 114°11'45.6"E, 780 m, 2.VIII.2014, Ke-ke Liu et al. leg.; 1 ♀, Ciping Town, Xiaojing Village, 26°35'20.4"N, 114°8'13.2"E, 913 m, 2.VIII.2014, K. Liu et al. leg.; 1 ♀, Ciping Town, Dajing Village, General of forest, 26.566°N, 114.125°E, 922 m, 13.VII.2014, K. Liu et al. leg.; 1 ♂, 1 ♀, Ciping Town, Dajing Village, 26°33'21.70"N, 114°07'20.08"E, 906 m, 31.VII.2019, K. Liu et al. leg.; 1 ♀, Dongshang Town, Jiangshan Village, Qilichuan, 26°46'18.91"N, 113°51'55.59"E, 666 m, 30.VII.2019, other data as previous; 1 ♀, 26°46'23.73"N, 113°52'02.83"E, 665 m, K. Liu et al. leg.; 2 ♂, Longshi Town, Bashang Village, 26°39'58.29"N, 114°04'35.34"E, 491 m, 29.VII.2019, K. Liu et al. leg.; 1 ♀, Suichuan County, Gaoping Town, Gaoping Bus Station, 26°02'49.6"N, 114°07'2.8"E, 482 m, 1.VIII.2019, K. Liu et al. leg.; 1 ♀, Ciping Town, Wuzhifeng Scenic Spot, 26°32'48.23"N, 114°09'10.61"E, 811 m, 2.X.2018, K. Liu et al. leg.

###### Comments.

The female of this species has remained undescribed till now and the male was confused with *X.kurilenis* Strand, 1907 in the past until Marusik and Omelko (2014) revealed some differences between these two species based on comparisons of the holotype of *X.lesserti* and specimens of *X.kurilenis* from the Kuril Islands. Several specimens of both sexes of *X.lesserti* were collected in the Jinggang Mountain National Nature Reserve including one pair which was in copula. Thus, collected females are considered by us as belonging to this species.

###### Diagnosis.

**Figure 14.**
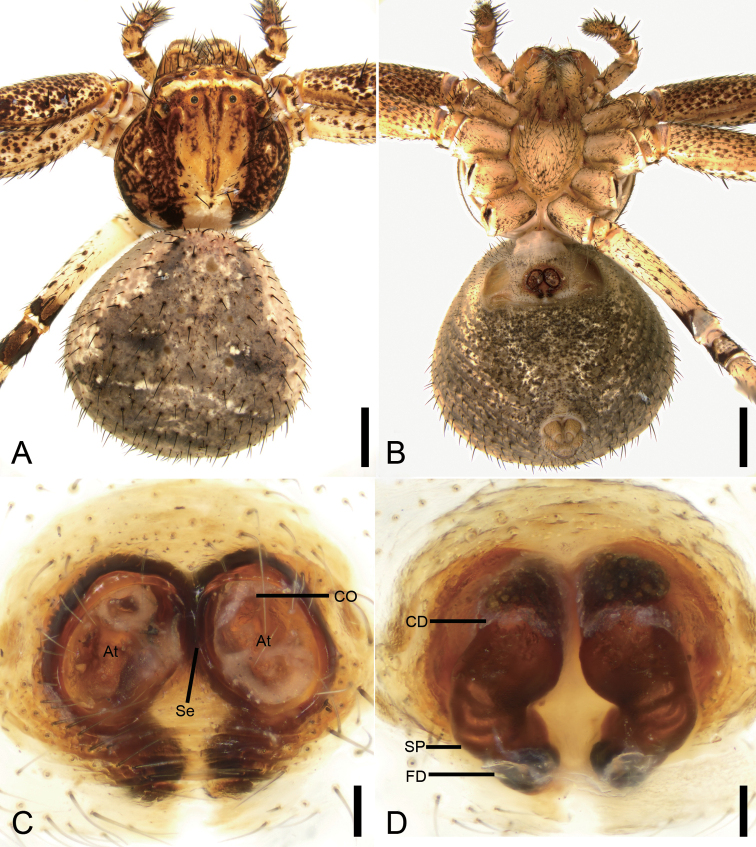
*Xysticuslesserti* Schenkel, 1963, female. **A** habitus, dorsal view **B** same, ventral view **C** epigyne, ventral view **D** same, dorsal view. Abbreviations: At – atrium, CD – copulatory duct, CO – copulatory opening, FD – fertilisation duct, Se –septum, SP – spermatheca. Scale bars: 1 mm (**A, B**), 0.1 mm (**C, D**).

**Figure 15.**
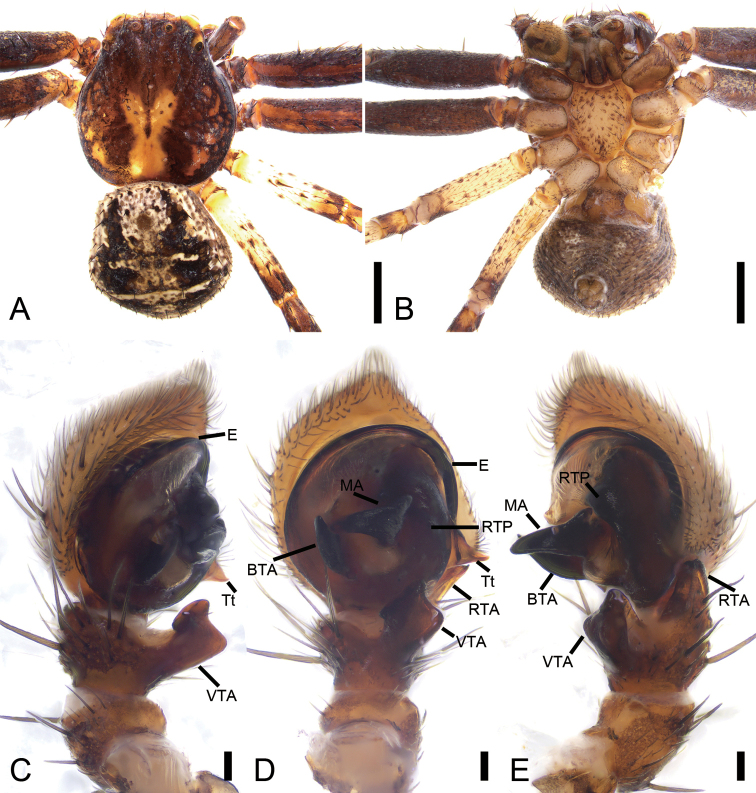
*Xysticuslesserti* Schenkel, 1963, male. **A** habitus, dorsal view **B** same, ventral view **C** palp, prolateral view **D** same, ventral view **E** same, retrolateral view. Abbreviations: BTA – basal tegular apophysis, E – embolus, MA – median apophysis, RTA – retrolateral tibial apophysis, RTP – ridge-shaped tegular process, Tt – tutaculum, VTA – ventral tibial apophysis. Scale bars: 1 mm (**A, B**), 0.1 mm (**C–E**).

The female of this species is similar to that of *X.kurilensis* in having two large oval atria (*At*) divided by septum (*Se*). Female of *X.lesserti* can be differentiated from those of sibling species by touching atria (vs. not touching) (cf. Fig. 14C and Kim and Lee 2012: fig. 21–4H). The male of *X.lesserti* is also very similar to those of *X.kurilensis*, but can be distinguished by the median apophysis (*MA*) reaching the sub-apex of the basal tegular apophysis (*BTA*) in ventral view (vs. not reaching the sub-apex of the conductor) (cf. Fig. 15D and Marusik and Omelko 2014: fig. 18).

###### Description.

**Female.** Habitus as in Fig. 14A, B. Total length 4.23. Carapace: 1.82 long, 1.81 wide. Eye sizes and interdistances: AME 0.12, ALE 0.24, PME 0.12, PLE 0.24, AME–AME 0.48, AME–ALE 0.28, PME–PME 0.51, PME–PLE 0.56, AME–PME 0.39, AME–PLE 0.87, ALE–ALE 1.25, PLE–PLE 1.82, ALE–PLE 0.39. MOA 0.62 long, front width 0.71, back width 0.77. Chelicerae toothless. Abdomen (Fig. 12A, B): 2.05 long, 2.19 wide, with numerous depressed patches. Leg measurements: I 5.39 (1.71, 0.81, 1.28, 1.05, 0.54); II 5.59 (1.70, 0.87, 1.30, 1.12, 0.6); III 3.36 (1.18, 0.44, 0.79, 0.55, 0.40); IV 4.03 (1.17, 0.60, 0.95, 0.75, 0.56). Leg spination: I Fe: d1, p4; Ti: p3, v11; Mt: p3, r2, v11; II Fe: d1; Ti: p3, r1, v10; Mt: p3, r2, v10; III Fe: d1; Ti: p2, v5; Mt: p2, r1, v4; IV: Fe: d1; Pa: d2; Ti: d1, p2, v2; Mt: p2, r1, v3.

Colouration (Fig. 14A, B). Carapace reddish brown with dark mottling, medially with broad pale yellow stripe extending from PME to posterior edge. Chelicerae, endites, and labium reddish yellow. Sternum yellow, with numerous dark brown spots. Legs: I and II darker than III and IV; femora I–IV dorsally with reddish brown stripes, other segments yellowish to dark brown, with dark brown spots. Abdomen with grey oval patten, sub-medially with two indistinct transverse whitish stripes.

Epigyne (Fig. 14C, D). Epigyne approximately as long as wide. Anteriorly with two atria (*At*) separated by the septum (*Se*). Copulatory openings (*CO*) located in the anterior part of atria, hidden by sclerotised anterior margins of atria. Spermathecae (*SP*) kidney-shaped, with several constrictions. Fertilisation duct (*FD*) short, as long as width of the spermatheca in its posterior part, directed laterally.

**Male.** Habitus as in Fig. 15A, B. As in female, except as noted. Total length 3.07. Carapace: 1.80 long, 1.56 wide, with a few strong setae around eye area. Eye sizes and interdistances: AME 0.12, ALE 0.27, PME 0.13, PLE 0.22, AME–AME 0.36, AME–ALE 0.22, PME–PME 0.39, PME–PLE 0.46, AME–PME 0.29, AME–PLE 0.71, ALE–ALE 0.99, PLE–PLE 1.30, ALE–PLE 0.35. MOA 0.51 long, front width 0.58, back width 0.69. Abdomen (Fig. 15A, B): 1.40 long, 1.43 wide, with abundant strong setae dorsally. Leg measurements: I 5.56 (1.66, 0.79, 1.13, 1.30, 0.68); II 5.36 (1.62, 0.70, 1.22, 1.20, 0.62); III 3.36 (1.18, 0.44, 0.79, 0.55, 0.40); IV 3.83 (1.17, 0.42, 0.94, 0.79, 0.51). Leg spination: I Fe: d5, p3; Pa: v1; Ti: p3, r3, v10; Mt: p3, r2, v8; II Fe: d5, p3; Pa: v1; Ti: p3, r3, v10; Mt: p3, r2, v10; III Fe: d4; Pa: d2, v1; Ti: d2, p2, r2, v6; Mt: p3, r3, v4; IV: Fe: d4; Pa: d2, r1; Ti: d2, p2, r2, v6; Mt: p3, r3, v4.

**Figure 16.**
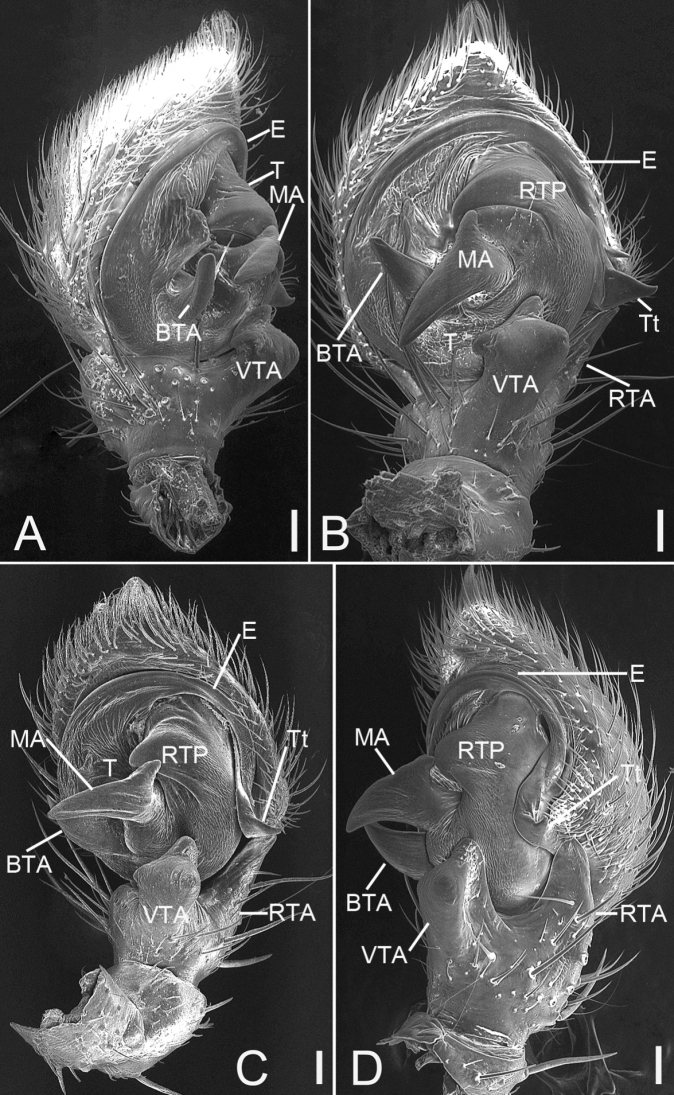
SEM micrographs of *Xysticuslesserti* Schenkel, 1963, male palp. **A** prolateral view **B** ventral view **C** ventral view, slightly retrolateral **D** retrolateral view. Abbreviations: BTA – basal tegular apophysis, E – embolus, MA – median apophysis, RTA – retrolateral tibial apophysis, RTP – ridge-shaped tegular process, T – tegulum, Tt – tutaculum, VTA – ventral tibial apophysis. Scale bars: 0.1 mm.

Palp (Figs 15C–E, 16). Tibia with two apophysis: the retrolateral one (*RTA*) triangular, shorter than tibia, the ventral one (*VTA*) square, longer than tibia. Cymbium irregularly oval, length/width ratio ~ 1.2. Tutaculum (*Tt*) triangular, forming a canal. Median apophysis (*MA*), strongly sclerotised, wing-shaped, its apex reaching the sub-apex of basal tegular apophysis (*BTA*). Basal tegular apophysis broad and stocky. Tegulum with a ridge-shaped retrolateral process (*RTP*). Base of the embolus (*E*) gradually separating from the tegulum, slightly tapering during its median portion, apex embedded in tutaculum.

###### Distribution.

**Figure 17.**
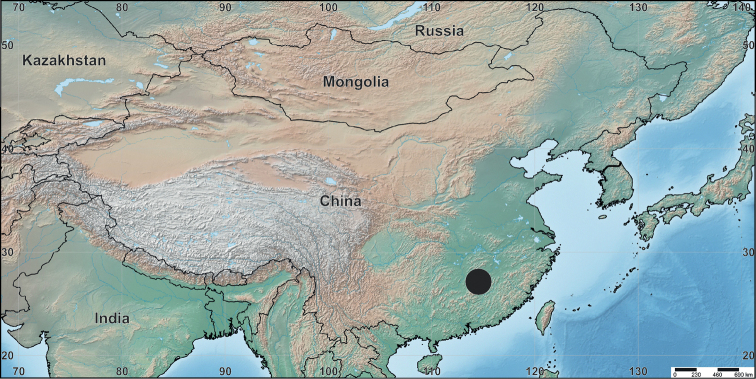
The location of the Jinggang Mountain National Nature Reserve in China indicated by a large black dot.

China: Jiangxi (Fig. 17) and Guizhou Provinces (Schenkel 1963); Russia: Sakhalin Is., Kurile Isles (Marusik and Omelko 2014); Korea and Japan (Songand Zhu 1997).

## Supplementary Material

XML Treatment for Angaeus

XML Treatment for Angaeus xieluae

XML Treatment for Ebelingia

XML Treatment for Ebelingia forcipata

XML Treatment for Lysiteles

XML Treatment for Lysiteles subspirellus

XML Treatment for Oxytate

XML Treatment for Oxytate bicornis

XML Treatment for Oxytate mucunica

XML Treatment for Pharta

XML Treatment for Pharta lingxiufengica

XML Treatment for Stephanopis

XML Treatment for Stephanopis xiangzhouica

XML Treatment for Xysticus

XML Treatment for Xysticus lesserti

## References

[B1] Benjamin SP (2011) Phylogenetics and comparative morphology of crab spiders (Araneae: Dionycha, Thomisidae). Zootaxa 3080(1): 1–108. 10.11646/zootaxa.3080.1.1

[B2] Benjamin SP (2013) On the crab spider genus *Angaeus* Thorell, 1881 and its junior synonym *Paraborboropactus* Tang and Li, 2009 (Araneae: Thomisidae). Zootaxa 3635(1): 71–80. 10.11646/zootaxa.3635.1.7 26097932

[B3] Dondale CD Redner JH (1978) The insects and arachnids of Canada, Part 5. The crab spiders of Canada and Alaska, Araneae: Philodromidae and Thomisidae. Research Branch Agriculture Canada Publication 1663: 1–255.

[B4] Kim JP Gwon SP (2001) A revisional study of the spider family Thomisidae Sundevall, 1833 (Arachnida: Araneae) from Korea. Korean Arachnology 17(1): 13–78.

[B5] Kim ST Lee SY (2012) Arthropoda: Arachnida: Araneae: Thomisidae. Thomisid spiders. Invertebrate Fauna of Korea 21(9): 1–88.

[B6] Li S (2020) Spider taxonomy for an advanced China. Zoological Systematics 45(2): 73–77.

[B7] Li SQ Lin YC (2016) Species Catalogue of China. Vol. 2. Animals. Invertebrates (1). Arachnida: Araneae. Science Press, Beijing, 549 pp.

[B8] Liu K Liu J Xu X (2017) Two new species of the genus *Oxytate* from China (Araneae: Thomisidae). Zootaxa 4320(1): 193–200. 10.11646/zootaxa.4320.1.12

[B9] Logunov DV (1992) On the spider fauna of the Bolshekhekhtsyrski State Reserv (Khabarovsk Province). I. Families Araneidae, Lycosidae, Philodromidae, Tetragnathidae and Thomisidae. Sibirskij Biologichesky Zhurnal 4: 56–68.

[B10] Machado M Teixeira RA (2021) Phylogenetic relationships in Stephanopinae: systematics of *Stephanopis* and *Sidymella* based on morphological characters (Araneae: Thomisidae). Organisms, Diversity & Evolution 21(2): 281–313. 10.1007/s13127-020-00472-x

[B11] Machado M Teixeira RA Milledge GA (2019) On the Australian bark crab spider genus *Stephanopis*: taxonomic review and description of seven new species (Araneae: Thomisidae: Stephanopinae). Records of the Australian Museum 71(6): 217–276. 10.3853/j.2201-4349.71.2019.1698

[B12] Marusik YM Omelko MM (2014) Reconsideration of *Xysticus* species described by Ehrenfried Schenkel from Mongolia and China in 1963 (Araneae: Thomisidae). Zootaxa 3861(3): 275–289. 10.11646/zootaxa.3861.3.5 25283408

[B13] Ono H (2001) Crab spiders of the family Thomisidae from the Kingdom of Bhutan (Arachnida, Araneae). Entomologica Basiliensis 23: 203–236.

[B14] Ono H (2009) The spiders of Japan with keys to the families and genera and illustrations of the species. Tokai University Press, Kanagawa, 739 pp.

[B15] Ramírez MJ (2014) The morphology and phylogeny of dionychan spiders (Araneae: Araneomorphae). Bulletin of the American Museum of Natural History 390: 1–374. 10.1206/821.1

[B16] Schenkel E (1963) Ostasiatische Spinnen aus dem Muséum d’Histoire naturelle de Paris. Mémoires du Muséum National d’Histoire Naturelle de Paris (A, Zool.) 25: 1–481.

[B17] Song DX Zhu MS (1997) *Fauna Sinica*: *Arachnida*: *Araneae*: *Thomisidae*, *Philodromidae*. Science Press, Beijing, 259 pp.

[B18] Song DX Zhu MS Li SQ (1993) Arachnida: Araneae. In: Huang CM (Ed.) Animals of Longqi Mountai.China Forestry Publishing House, Beijing, 852–890.

[B19] Song DX Zhu MS Chen J (1999) The spiders of China. Hebei Science and Technology Publishing House, Shijiazhuang, 640 pp.

[B20] Tang G Li SQ (2010a) Crab spiders from Hainan Island, China (Araneae, Thomisidae). Zootaxa 2369(1): 1–68. 10.11646/zootaxa.2369.1.1

[B21] Tang G Li SQ (2010b) Crab spiders from Xishuangbanna, Yunnan Province, China (Araneae, Thomisidae). Zootaxa 2703(1): 1–105. 10.11646/zootaxa.2703.1.1

[B22] Tang G Yin CM Peng XJ Ubick D Griswold C (2008) The crab spiders of the genus *Lysiteles* from Yunnan Province, China (Araneae: Thomisidae). Zootaxa 1742(1): 1–41. 10.11646/zootaxa.1742.1.1

[B23] Wang C Mi XQ Peng XJ (2016) A new species of *Pharta* Thorell, 1891 (Araneae: Thomisidae) from China. Oriental Insects 50(3): 129–134. 10.1080/00305316.2016.1197163

[B24] World Spider Catalog (2022) World Spider Catalog. Natural History Museum Bern. Version 22.5. https://wsc.nmbe.ch/ [accessed 2 January 2022]

